# Prospective Assessment of Changes in Target Vessel Peak Systolic Velocity Measurements After Fenestrated-Branched Endovascular Aortic Repair

**DOI:** 10.1177/15266028251368250

**Published:** 2025-09-28

**Authors:** Titia A. L. Sulzer, Thanila A. Macedo, Thomas Mesnard, Emanuel R. Tenorio, Guilherme Baumgardt Barbosa Lima, Heather Hatz, Gina K. Hesley, Alexander Lekah, Tiziano Tallarita, Ying Huang, Bernardo C Mendes, Gustavo S. Oderich

**Affiliations:** 1 Department of Cardiothoracic and Vascular Surgery, McGovern Medical School, Advanced Aortic Research Program at the University of Texas Health Science Center at Houston, Houston, USA; 2 Department of Vascular and Endovascular Surgery, Mayo Clinic, Rochester, MN, USA; 3 Department of Radiology, Mayo Clinic, Rochester, MN, USA; 4 Department of Vascular Surgery, Mayo Clinic, Eau Claire, WI, USA

**Keywords:** duplex ultrasound, complex abdominal aortic aneurysms, thoracoabdominal aortic aneurysm, peak systolic velocity, stenosis

## Abstract

**Objective:**

Target vessel stenosis or occlusion is a common indication for secondary intervention after fenestrated-branched endovascular aortic repair requiring long-term imaging surveillance including duplex ultrasound (DUS). This study aimed to describe and compare longitudinal changes in peak systolic velocity (PSV) measurements for renal and mesenteric arteries targeted by directional branches (DBs) or reinforced fenestrations (RFs).

**Methods:**

Patients enrolled in a prospective, nonrandomized study (2013-2020) had DUS of celiac axis, superior mesenteric artery, and renal arteries (RAs) obtained preoperatively, at 6 to 8 weeks, 6 months, and annually. Vessels with preprocedural stenosis were excluded. Outcomes were variations in PSV over time for target vessels incorporated by DBs or RFs, differences in balloon-expandable stent-grafts (BESGs) and self-expandable stent-grafts (SESGs), PSV measurements prior to secondary interventions related to vessel stenosis, and a predictive value of PSV for stenosis requiring secondary intervention.

**Results:**

A total of 419 patients (292 male, mean age 74 ± 8 years old) were enrolled, with 1,311 target vessels analyzed preoperatively, including 607 mesenteric and 704 RAs. Over a median follow-up of 23 months (interquartile range [IQR], 7-36), PSV measurements decreased in the first 6 to 8 weeks after DB incorporation, remained stable, followed by a nonsignificant increase at 5 years. PSV increased in the first 6 months using RFs with postoperative velocities significantly higher (*P* < .05) for RFs compared to DBs. Branched mesenteric vessels stented with BESGs had higher velocities than SESGs (*P* < .05). Of the 23 target vessels treated by secondary intervention for stenosis, 19 (83%) had velocities above the thresholds for native, nonstented vessels. Furthermore, in RAs (n = 20) PSV effectively predicted clinically relevant stenosis, AUC was 0.98, with a 231 cm/s threshold offering 84% sensitivity with 100% specificity.

**Conclusion:**

Longitudinal follow-up shows that velocity changes vary depending on the type of incorporation, vessel, and bridging stent. For DBs, PSV decreased initially, stabilized, and showed a nonsignificant increase at 5 years. In contrast, PSV increased with RFs and remained higher than DBs. Despite these variations, velocities remained below established thresholds for clinically significant stenosis in nonstented vessels. Criteria for in-stent stenosis may differ, and PSV alone should not be the sole indicator for reintervention.

**Clinical Impact:**

This study provides new insight into duplex ultrasound (DUS) surveillance after fenestrated and branched endovascular aneurysm repair (FB-EVAR). Peak systolic velocity (PSV) trends differed between fenestrations and branches, and stent type influenced flow dynamics. Importantly, most vessels with secondary interventions had elevated PSVs, but many others exceeded native stenosis thresholds without clinical consequence. Despite these variations, velocities remained below established thresholds for clinically significant stenosis in non-stented vessels. Criteria for in-stent stenosis may differ, and PSV alone should not be the sole indicator for reintervention.The reported PSV measurements served as a benchmark for DUS surveillance following FB-EVAR.

## Introduction

Fenestrated and branched endovascular aortic aneurysm repair (FB-EVAR) has expanded the scope and applicability of endovascular techniques in the treatment of complex aortic aneurysms. The technique encompasses variations of stent designs with reinforced fenestrations (RFs) or directional branches (DBs) to incorporate the renal and mesenteric vessels with the placement of bridging stent-grafts.^1,2^ Lifelong surveillance is required, as target vessel complications remain one of the most frequent indications for secondary interventions.^3,4^ According to data from the US Aortic Research Consortium, the rates for target artery occlusion or stenosis are 2% for mesenteric arteries and 8% for renal arteries (RAs).^5^

Despite computed tomographic angiography (CTA) serving as the primary method for surveillance after FB-EVAR, duplex ultrasound (DUS) is a valuable complementary noninvasive imaging modality, detecting hemodynamic alterations in stents. This is especially useful in small, stented vessels, where metallic artifact limits accuracy of CTA evaluation.^6,7^ The 2 modalities, CTA and DUS, are often complementary for decision-making into timing and type of secondary intervention if needed.

Ultrasound is a desirable modality because of its favorable cost, wide availability, no need for iodinated contrast, and absence of ionizing radiation.^7^ DUS examination after FB-EVAR requires qualified sonographers, preferentially trained in vascular imaging, to maximize success and accuracy of results. Although the ability to detect vessel stenosis or occlusion is a valuable advantage of DUS, morphologic complications such as migration and stent fracture remain a limitation of current ultrasound techniques.^8,9^ A prior report by our group confirmed that stenting of nonstenotic renal and mesenteric arteries during FB-EVAR is not associated with increase in target vessel peak systolic velocity (PSV) in the first 6 to 8 weeks.^10^ Nonetheless, there is paucity of evidence concerning the long-term effects of stenting nonstenotic vessels following FB-EVAR, as well as which threshold should be used for reintervention, if any. The aim of this study was to assess velocity measurements over time after FB-EVAR and describe velocity measurements among patients who underwent secondary interventions for stent stenosis.

## Methods

### Study Design and Population

This study involved a cohort of patients enrolled in an ongoing prospective nonrandomized physician-sponsored investigational device exemption protocol (Clinical Trial number: NCT02089607) to evaluate FB-EVAR between October 2013 and May 2020. Eligible patients included those undergoing FB-EVAR for complex aortic aneurysms. The study was approved by the Institutional Review Board and patients provided informed consent. Prospective DUS evaluations of renal-mesenteric vessels were conducted before the procedure, at 6 to 8 weeks after the procedure, 6 months, 1 year, and yearly thereafter for the first 5 years. The examinations were all performed in a single ultrasound laboratory with a high volume of vascular ultrasound examinations (over 10 000 studies per year). These were conducted by registered vascular technologist (RVTs), with several years of experience and a substantial vascular exam-case load, all following a standardized protocol. We excluded vessels with significant stenosis defined preoperatively by DUS, according to established criteria of ≥60% stenosis in the RAs (200 cm/s) and ≥70% stenosis in the celiac axis (CA) and superior mesenteric artery (SMA; 200 and 275 cm/s, respectively), to eliminate potential confounding factors in assessing velocity changes specifically related to stent placement.^11-13^ This study followed the relevant Strengthening of the Reporting of Observational Studies in Epidemiology (STROBE) guidelines.^14^

### Outcomes

The study’s primary outcome was to assess the variations in PSV (cm/s) of the stented mesenteric and RAs over a 5-year period, stratified by a type of incorporation (RFs and DBs). Secondary outcomes included changes in PSV (cm/s) over a 5-year period for target vessels incorporated with DBs using either balloon-expandable stent-grafts (BESGs) or self-expandable stent-grafts (SESGs), a description of PSV measurements obtained prior to a secondary intervention related to vessel stenosis, and an exploratory analysis of the predictive value of PSV for stenosis requiring secondary intervention.

### Definitions

For the analysis of secondary intervention related to vessel stenosis, we excluded vessels without DUS examination immediately prior to the secondary intervention. The indication for a secondary intervention was individualized and left at the discretion of the primary investigator (G.S.O.). Factors considered in the decision to intervene included perceived severity of stenosis after analysis of PSV and CTA, possible underlying etiology, progression rate, and other comorbidities (eg, chronic kidney disease, and solitary kidney). This also applied to some asymptomatic patients, in whom high-grade stenosis or high-risk clinical context justified intervention. Furthermore, patient demographics, comorbidities, cardiovascular risk factors, medication status, aortic history, procedural outcomes, and anatomic and device characteristics were collected. Definitions were based on the Society for Vascular Surgery (SVS) reporting standards for endovascular repair of aneurysms involving the renal and mesenteric arteries.^15^

### Endovascular Technique

Patients received either a patient-specific stent graft with any combination of RFs and/or DBs or an off-the-shelf multibranch stent graft manufactured by Cook Medical (Bloomington, IN, USA). The selection of the stent-graft design was performed at the discretion of the principal investigator, taking into consideration specific anatomical characteristics of the target vessel, for example, factors such as the extent of the aneurysm, the inner luminal diameter of the aorta, and the orientation of the target vessel. Fenestrations were aligned with BESGs, whereas branches were aligned with either BESGs or SESGs based on anatomical considerations and operator preferences. The procedural techniques have been described previously.^16^

### DUS Protocol

DUS scans were performed using GE E9 scanners by experienced RVTs. The DUS protocol has been described in detail by our team in a prior study.^10^ DUS was performed using predefined protocols prospectively and included thorough PSV (cm/s) measurements obtained with <60° angles throughout the visualized vessel.

### Statistical Analysis

For the demographics, baseline comorbidities, and anatomic and device characteristics, we used descriptive statistics (counts and percentages only), without inferential testing. The continuous variable PSV was evaluated for normal distribution using histograms and Q–Q plots and reported as mean and standard deviation. Mesenteric arteries and RAs were evaluated independently. We used the maximum of the segments (origin, proximal, and mid) of all target vessels in our longitudinal analysis. For the threshold of PSV at the time of secondary interventions for stenosis, the highest PSV value of the segments of each vessel was used, and the median and interquartile range (IQR) were reported. *T*-tests were used to perform pairwise comparisons of velocities between consecutive time points (baseline to 5 years) to evaluate differences. To illustrate the changes in measured PSVs at different time points within the mesenteric and RAs, as well as between SESGs and BESGs, we used spaghetti plots. These plots were supplemented with locally weighted scatter plot smoothing regression lines (LOESS) and with 95% confidence intervals (95% CI). LOESS is a nonparametric regression method effect that uses locally weighted regression to fit a smooth curve through points in a scatterplot. This method showed the estimated overall variability of the PSVs.^17^ Analysis of variance (ANOVA) was chosen to assess the effects of the independent variables (DBs vs RFs and BESGs vs SESGs) on the dependent variable (PSV) across multiple time points. To evaluate the predictive value of PSV for stenosis requiring secondary intervention, a 1:1 nearest-neighbor matching of stenotic target vessels with nonstenotic controls was performed, based on vessel type, incorporation, and follow-up duration. Subsequently, a receiver operating characteristic (ROC) analysis was conducted, and the optimal PSV threshold was determined using Youden’s index.

A *P*-value of less than .05 was considered statistically significant. The data analysis was performed using R version 4.0.3 (R Foundation for Statistical Computing, Vienna, Austria).

## Results

### Patient Characteristics

There were 419 patients, 292 male and 127 female, with mean age of 74 ± 8 years-old treated by FB-EVAR between October 2013 and May 2020 who were included in the analysis. Aneurysm extent was classified as thoracoabdominal aortic aneurysm (TAAA) in 69% (Crawford extent IV in 31%) or complex abdominal aortic aneurysms in 31%. The mean number of renal-mesenteric vessels targeted per patient was 3.9 ± 0.5. Demographic information, baseline comorbidities, and anatomic details are listed in Table 1.

**Table 1.** Demographics and Anatomical Characteristics of 419 Patients Treated by FB-EVAR for TAAA or Pararenal Aortic Aneurysms.

Demographics	n = 419
Gender, male	292 (70)
Gender, female	127 (30)
Age, years	74 ± 8.1
BMI	28 ± 5.5
Comorbidities	
Coronary artery disease	207 (49)
CHF	37 (8.8)
Diabetes	57 (14)
Hypertension	371 (86)
Hypercholesterolemia	331 (79)
COPD	127 (30)
Tobacco use	330 (79)
PAD	65 (16)
Stroke/TIA	42 (10)
CKD	195 (47)
Anatomical characteristics	
Maximum aortic aneurysm diameter	65.1 ± 10.9
No. of target vessels per patient	3.89 ± 0.52
Crawford extent	
Extent I	20 (4.8)
Extent II	97 (23)
Extent III	43 (10)
Extent IV	131 (31)
Pararenal	128 (31)
Aneurysm status	
Asymptomatic	411 (98)
Symptomatic nonruptured	6 (1.4)
Contained rupture	2 (0.5)

Abbreviations: BMI, body mass index; CHF, congestive heart failure; CKD, chronic kidney disease; COPD, chronic obstructive pulmonary disease; PAD, peripheral arterial disease; TAAA, thoracoabdominal aortic aneurysm; TIA, transient ischemic attack.

Data are presented as n (%) or mean ± standard deviation.

Significant stenosis was identified in 161 targeted vessels preoperatively, and these vessels were subsequently excluded from the study. The remaining 1411 targeted vessels, including 260 CAs, 409 SMAs, and 742 RAs, had no evidence of preexisting underlying stenosis.

**Figure 1.**
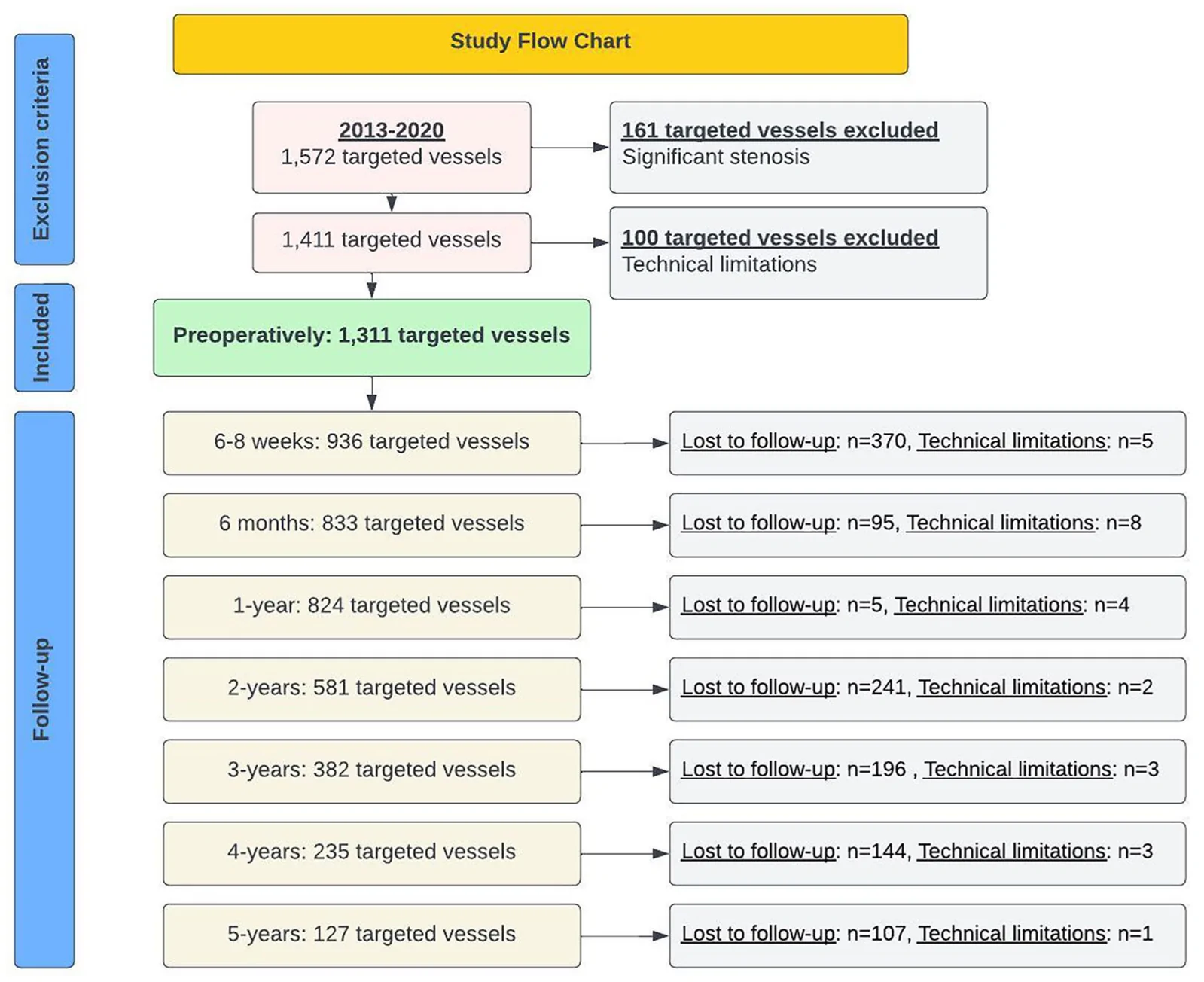
Study flowchart showing the number of target vessels excluded preoperatively, lost to follow-up, or not evaluated due to technical issues.

Preoperatively, 100 vessels could not be visualized due to technical limitations (body habitus and overlying bowel gas; CA: n = 42; SMA: n = 29; RAs: n = 29). The study included 1311 vessels identified by ultrasound preoperatively and followed over a 5-year study period, with some patients having missed or incomplete follow-up DUS studies (Figure 1).

The overall technique success rate for ultrasound evaluations was 98% with 126/5229 vessels failed to obtain a measurement due to technique limitations including overlying bowel gas, body habitus, limited scanning window, and uncooperative patient. In 19 vessels, relining with additional covered stents was performed, based on the clinical decision of the primary investigator (G.S.O.). Bare metal stent relining was not performed in our cohort.

### Changes in PSV Over Time Between RFs and DBs in Mesenteric Arteries

**Figure 2.**
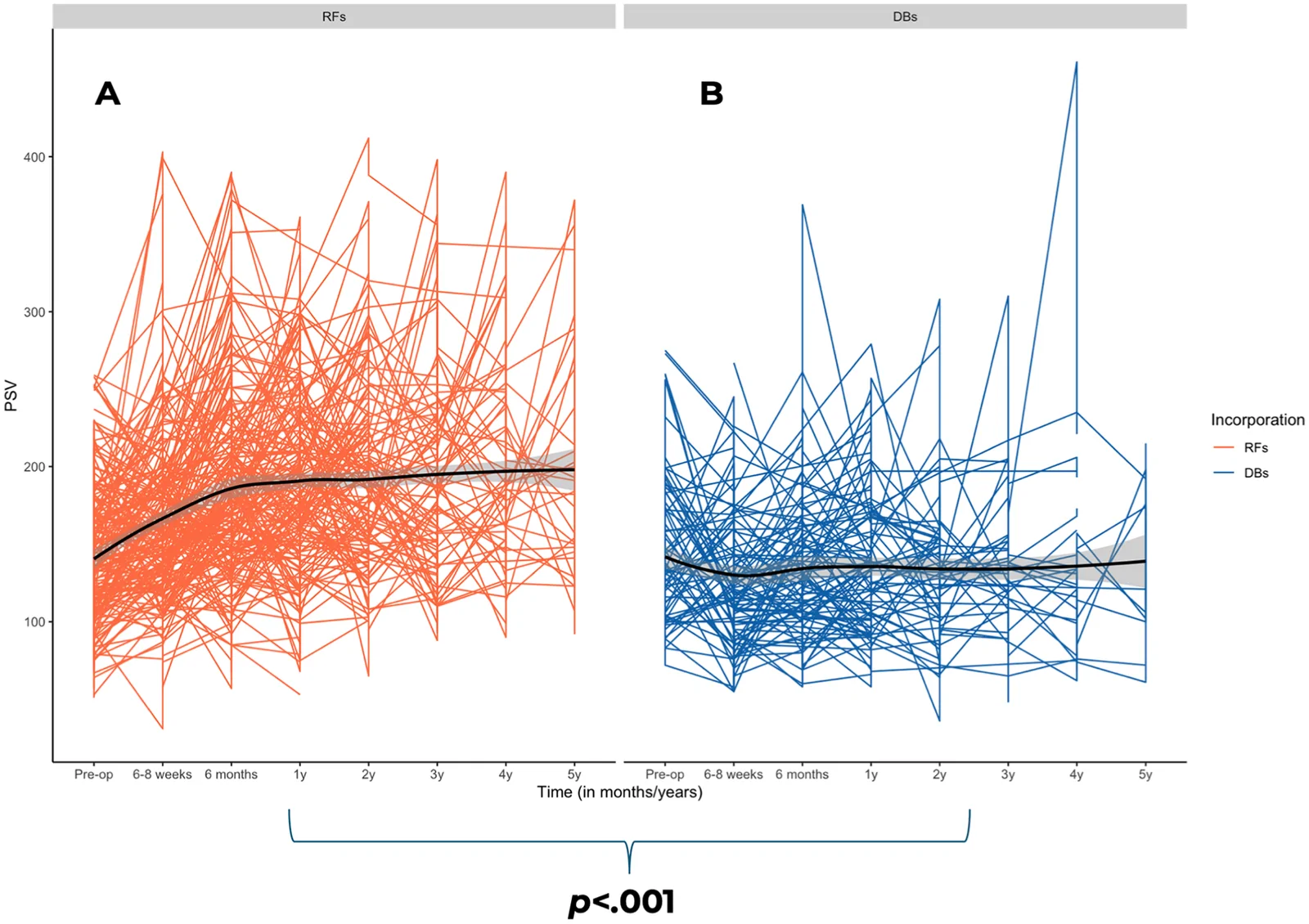
Changes in maximum PSV over time between reinforced fenestrations (A) and directional branches (B) in mesenteric arteries following fenestrated-branched endovascular aortic repair.﻿ PSV, peak systolic velocity.

In mesenteric arteries following FB-EVAR, DBs, and RFs had a significant impact on the PSV over a 5-year period (ANOVA-test, *P* < .001; Figure 2). Among vessels targeted using RFs, PSV significantly increased during the first 6 months, followed by a continued, nonsignificant increase in PSVs until year 5 (Table 2). The maximum PSV was 148 ± 52 cm/s at baseline, 166 ± 53 cm/s at 6 to 8 weeks, 187 ± 62 cm/s at 6 months, 190 ± 58 cm/s at 1 year, 193 ± 60 cm/s at 2 years, 197 ± 64 cm/s at 3 years, 204 ± 67 cm/s at 4 years, and 198 ± 69 cm/s at 5 years. Conversely, vessels targeted by DBs showed a significant decrease in PSV in the first 6 to 8 weeks, followed by nonsignificant fluctuations with a gradual increase until year 1, a slight decrease by year 2, and another increase until year 5 (Table 2). Maximum PSV was 158 ± 67 cm/s at baseline, 127 ± 43 cm/s at 6 to 8 weeks, 135 ± 45 cm/s at 6 months, 137 ± 45 cm/s at 1 year, 132 ± 44 cm/s at 2 years, 134 ± 44 cm/s at 3 years, 135 ± 69 cm/s at 4 years, and 139 ± 44 cm/s at 5 years. Velocities did not differ significantly between male and females.

**Table 2.** Pairwise Comparisons of Maximum Peak Systolic Velocity in Mesenteric Arteries After FB-EVAR, Including RFs and DBs, at Consecutive Time Points (Baseline to 5 Years).

Mean (SD)	RFs (cm/s)	*P*-value	DBs (cm/s)	*P*-value
Baseline	148 ± 52		158 ± 67	
6-8 Weeks	166 ± 53	**<.001**	127 ± 43	**<.001**
6 Months	187 ± 62	**<.001**	135 ± 45	.12
1 Year	190 ± 58	.30	137 ± 45	.96
2 Years	193 ± 60	.60	132 ± 44	.09
3 Years	197 ± 64	.86	134 ± 44	.18
4 Years	204 ± 67	.58	135 ± 69	.71
5 Years	198 ± 69	.62	139 ± 44	.11

Abbreviations: DBs, directional branches; RFs, reinforced fenestrations; SD, standard deviation.

*P*-values calculated between time intervals. Values in bold indicate statistical significance (*p* < 0.05).

### Changes in PSV Over Time Between RFs and DBs in RAs

**Figure 3.**
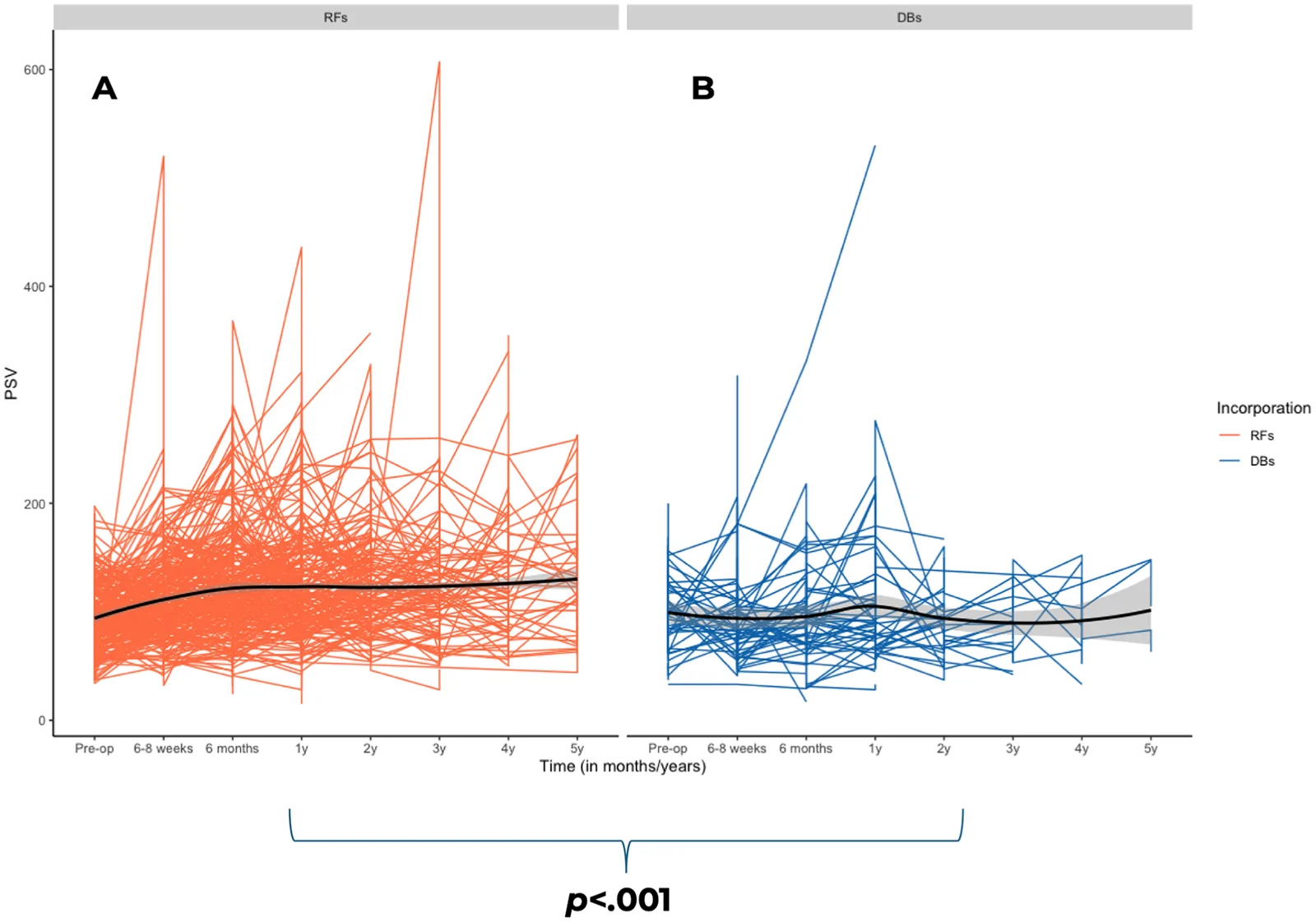
Changes in maximum PSV over time between reinforced fenestrations (A) and directional branches (B) in renal arteries following fenestrated-branched endovascular aortic repair. PSV, peak systolic velocity.

For RAs, DBs and RFs also significantly affected the PSV over a 5-year period (ANOVA-test, *P* < .001; Figure 3). Unlike observations in mesenteric arteries, RFs showed a significant increase in PSV measurements from 6 to 8 weeks to 6 months, followed by stabilization with small, nonsignificant fluctuations until year 4, and a small but significant increase in year 5 (Table 3). Maximum PSV was 108 ± 59 cm/s at baseline, 108 ± 43 cm/s at 6 to 8 weeks, 118 ± 52 cm/s at 6 months, 118 ± 52 cm/s at 1 year, 120 ± 46 cm/s at 2 years, 117 ± 55 cm/s at 3 years, 122 ± 55 cm/s at 4 years and 131 ± 50 cm/s at 5 years. Conversely, DBs showed a significant decrease in PSV measurements in the first 6 to 8 weeks, followed by nonsignificant fluctuations, with a slight increase at 6 months to 1 year, a decrease by year 3, and another increase by year 5 (Table 3). Maximum PSV was 124 ± 79 cm/s at baseline, 94 ± 44 cm/s at 6 to 8 weeks, 95 ± 45 cm/s at 6 months, 104 ± 65 cm/s at 1 year, 91 ± 33 cm/s at 2 years, 82 ± 30 cm/s at 3 years, 87 ± 38 cm/s at 4 years, and 109 ± 38 cm/s at 5 years. Velocities did not differ significantly between male and females.

**Table 3.** Pairwise Comparisons of Maximum Peak Systolic Velocity in Renal Arteries After FB-EVAR, Including RFs and DBs, at Consecutive Time Points (Baseline to 5 Years).

Mean (SD)	RFs (cm/s)	*P*-value	DBs (cm/s)	*P*-value
Baseline	108 ± 59		124 ± 79	
6-8 Weeks	108 ± 43	.35	94 ± 44	.**019**
6 Months	118 ± 52	**<.001**	95 ± 45	.057
1 Year	118 ± 52	.26	104 ± 65	.080
2 Years	120 ± 46	.20	91 ± 33	.10
3 Years	117 ± 55	.43	82 ± 30	.56
4 Years	122 ± 55	.83	87 ± 38	.45
5 Years	131 ± 50	.**006**	109 ± 38	.43

Abbreviations: DBs, directional branches; RFs, reinforced fenestrations; SD, standard deviation.

*P*-values calculated between time intervals. Values in bold indicate statistical significance (*p* < 0.05).

### Changes in PSV Over Time Between BESGs and SESGs in Branched Mesenteric Arteries

**Figure 4.**
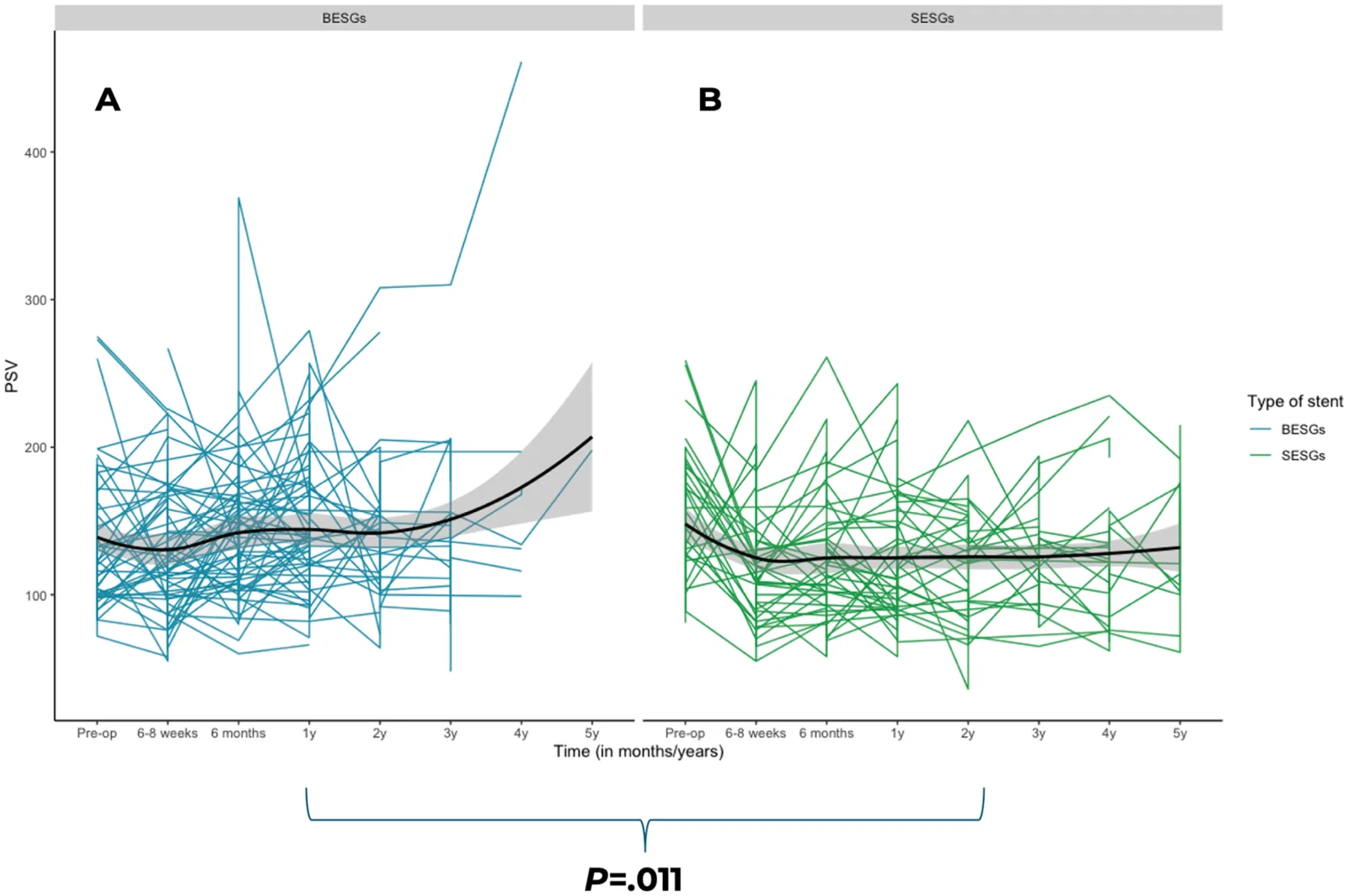
Changes in maximum PSV over time between balloon-expandable stent-grafts (A) and self-expandable stent-grafts (B) in branched mesenteric arteries following fenestrated-branched endovascular aortic repair. PSV, peak systolic velocity.

In total, there were 128 branched mesenteric arteries, of which 82 were BESGs and 46 were SESGs. In branched mesenteric arteries, BESGs and SESGs had a significant effect on the PSV (ANOVA-test, *P* = .011; Figure 4). BESGs exhibited a decrease from baseline to 6 to 8 weeks, followed by a nonsignificant increase that remained stable until year 3. Another increase occurred at years 4 and 5, although none of these changes were statistically significant. Maximum PSV for BESG was 157 ± 71 cm/s at baseline, 132 ± 46 cm/s at 6 to 8 weeks, 143 ± 47 cm/s at 6 months, 144 ± 45 cm/s at 1 year, 141 ± 49 cm/s at 2 years, 141 ± 54 cm/s at 3 years, 185 ± 116 cm/s at 4 years, and 198 ± NA cm/s at 5 years. Conversely, SESGs demonstrated a significant decrease from baseline to 6 to 8 weeks (*P* < .001), followed by a nonsignificant modest increase with velocities remaining below preoperative levels. Maximum PSV was 160 ± 57 cm/s at baseline, 119 ± 38 cm/s at 6 to 8 weeks, 126 ± 41 cm/s at 6m, 127 ± 43 cm/s at 1 year, 124 ± 38 cm/s at 2 years, 128 ± 34 cm/s at 3 years, 123 ± 45 cm/s at 4 years, and 136 ± 42 cm/s at 5 years.

### Fenestrated Mesenteric Arteries—BESGs Only

All RF target vessels were incorporated by BESGs; detailed PSV trends for these are reported in the section “Changes in PSV Over Time Between RFs and DBs in Mesenteric Arteries.” When comparing RFs and DBs incorporated by BESGs, the velocities for the RF group remained higher, confirming that higher velocities in the RF group cannot be justified only by the type of stent used.

### Changes in PSV Over Time Between BESGs and SESGs in Branched RAs

**Figure 5.**
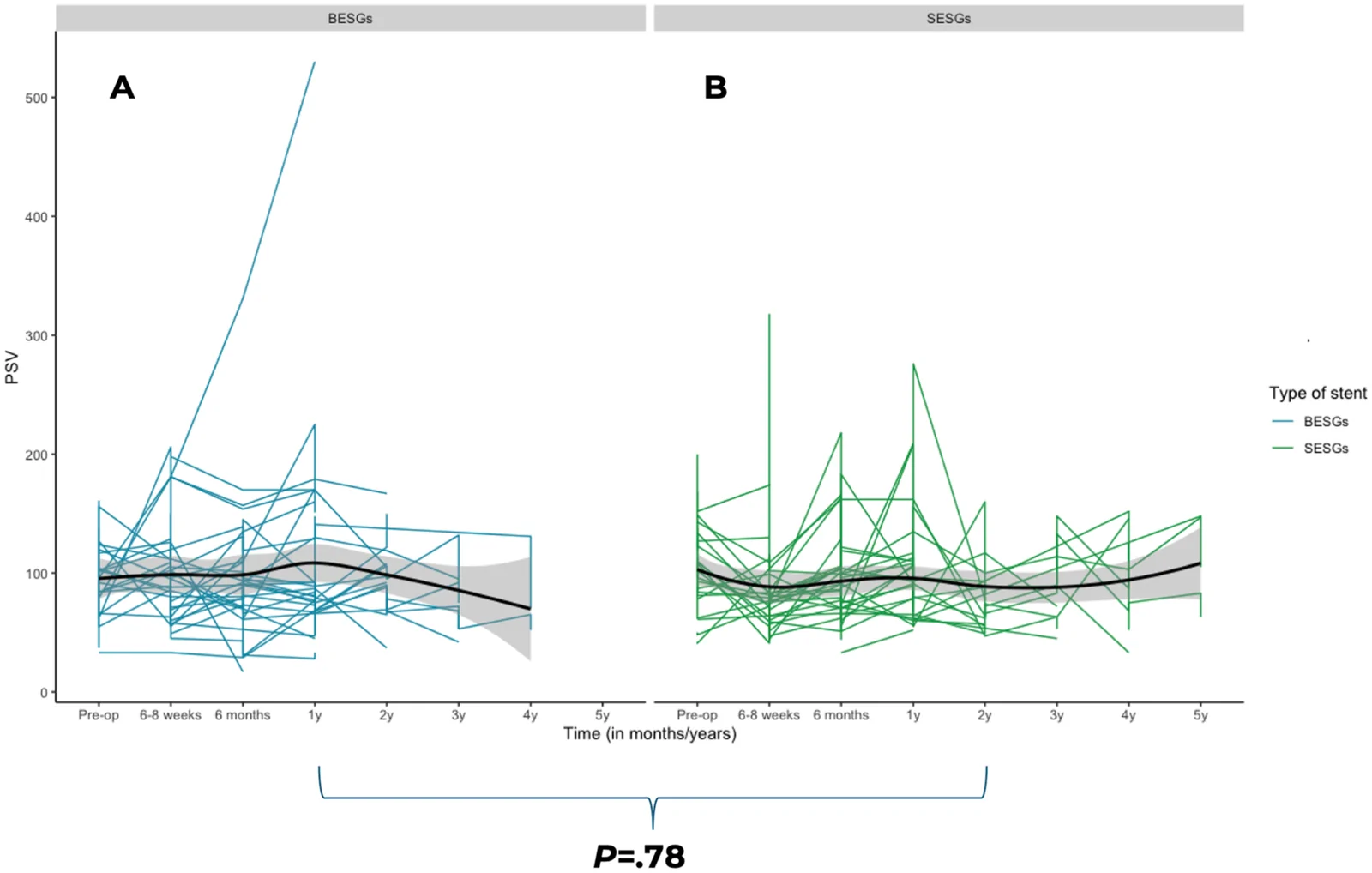
Changes in maximum PSV over time between balloon-expandable stent-grafts (A) and self-expandable stent-grafts (B) in branched renal arteries following fenestrated-branched endovascular aortic repair. PSV, peak systolic velocity.

In total, there were 72 branched RAs, of which 35 were BESGs and 37 were SESGs. In branched RAs, BESGs and SESGs did not have a significant effect on PSV (ANOVA-test, *P* = .78) (Figure 5). BESGs showed a nonsignificant decrease from baseline to 6 to 8 weeks, followed by nonsignificant fluctuations, but consistently stayed below baseline values. Maximum PSV was 124 ± 96 cm/s at baseline, 99 ± 41 cm/s at 6 to 8 weeks, 99 ± 54 cm/s at 6 months, 108 ± 78 cm/s at 1 year, 99 ± 34 cm/s at 2 years, 76 ± 27 cm/s at 3 years, and 80 ± 35 cm/s at 4 years. However, SESGs demonstrated a significant decrease from baseline to 6 to 8 weeks (*P* < .001) followed by an increase until 6 months (*P* = .005), followed by another significant decrease from year 1 to year 2 (*P* = .024), values stayed below baseline. Maximum PSV was 124 ± 61 cm/s at baseline, 88 ± 47 cm/s at 6 to 8 weeks, 92 ± 37 cm/s at 6m, 98 ± 48 cm/s at 1 year, 83 ± 30 cm/s at 2 years, 86 ± 31 cm/s at 3 years, 90 ± 41 cm/s at 4 years, and 109 ± 38 cm/s at 5 years.

### Threshold of PSV at Time of Secondary Interventions for Stenosis

During the study period, 31 of the 1311 target vessels included in the analysis required a secondary intervention for stenosis (DBs n = 10; RFs n = 21). Of these 31 vessels, 23 had a DUS examination prior to the intervention. This included 3 SMAs (1 DB, 2 RFs) and 20 RAs (9 DBs, 11 RFs). Notably, 11 underwent intraoperative relining with additional covered stents. The median PSV at time of intervention for SMAs was 350 cm/s ([IQR], 122-388 cm/s). For the RAs, the median was 308 cm/s ([IQR], 239-405 cm/s). DBs had lower PSVs for SMA (DBs [n = 1], 122 cm/s versus RFs [n = 2]; 350 and 388 cm/s) and a lower median PSV for RAs (276 vs 318 cm/s) as compared to RFs. Overall, 2 SMAs and 17 RAs had PSVs above the threshold of stenosis for native nonstented vessels (83%, 19/23). After propensity score matching, ROC analysis of 23 stenotic vessels and 23 control vessels was performed. In RAs (n = 20), PSV effectively predicted clinically relevant stenosis, AUC was 0.98, with a 231 cm/s threshold offering 84% sensitivity with 100% specificity. ROC analysis for mesenteric arteries (n = 3) was not feasible due to low numbers.

We also aimed to provide information on the number of vessels and patients that had maximum velocities above the threshold for stenosis, without requiring a secondary intervention. During the follow-up, 66 CAs, 36 SMAs, and 38 RAs had at some point maximum velocities above 200 and 275 cm/s.

## Discussion

This study prospectively evaluated longitudinal changes in DUS measurements after FB-EVAR, including differences in DBs and RFs and type of bridging stents. Over a 5-year period, PSV measurements decreased after target vessel incorporation using DBs and increased using RFs with postoperative velocities higher for RFs as compared to DBs. Moreover, the type of stent used for DBs also influenced the PSV over time. PSVs exceeding the recommended threshold for stenosis in native (nonstented) vessels were identified in 83% of target vessels at time of secondary intervention, indicating that this was not the sole criteria used to determine reinterventions for in-stent stenosis. Finally, the ROC analysis performed in RAs, gave an AUC of 0.98, indicating PSV effectively predicts clinically relevant stenosis. Thresholds of 231 cm/s gave 84% sensitivity and 100% specificity.

When hypothetically applying this new threshold to our cohort, an additional 16 RAs would have had interventions unnecessarily, instead of 38 RAs when using the threshold for native nonstented vessels (>200/s). While this represents an improvement, it would have resulted in 80% increase in the number of secondary interventions compared to what was actually performed (36 instead of 20) obviating the need for refinement and caution using this velocity threshold criteria. However, this analysis does not account for the natural history of vessels with elevated PSVs that did not undergo intervention. Thus, it remains uncertain whether these higher velocities truly represent clinically relevant stenoses that would have progressed or become symptomatic if left untreated. This highlights the need for prospective studies to validate these thresholds. Unfortunately, ROC analysis for mesenteric arteries was not feasible due to low numbers.

We found that incorporation with DBs in renal-mesenteric arteries causes a significant decrease of PSV in the first 6 to 8 weeks, with small nonsignificant changes over the 5 years remaining below the baseline velocities (Tables 2 and 3). This observation may correlate with changes in flow dynamics, which in nearly all cases was antegrade using caudally oriented branches. For RFs, mesenteric arteries have a significant increase in PSV in the first 6 months, which then stabilizes until year 5 (Table 2). For RFs in RAs, we found a significant increase from 6 to 8 weeks to 6 months, which remained stable until year 4, followed by a significant increase in year 5 (Table 3). Importantly, even though there are slight statistical differences between types of incorporation, these were not considered clinically relevant with mean PSVs still below the thresholds of clinically significant stenosis when the criteria for native nonstented vessels are used (Figures 2 and 3). These results add to the findings of our prior report which compared preoperative and early postoperative (6-8 weeks) measurements. In that study, DBs had a significant decrease in PSV and RFs showed a significant increase in PSV in most target vessel segments.^10^ According to our long-term findings in DBs, the initial decrease stabilizes after 6 to 8 weeks until year 4, with a small increase in year 5, although not significant. This descriptive trend could serve as a reference during follow-up, where deviations from this baseline pattern may indicate lesions that merit further imaging or reintervention. However, these findings are based on retrospective data and should be validation in larger prospective studies. We have also confirmed the velocity differences between DBs and RFs by comparing fenestrated mesenteric arteries, which are all incorporated by BESGs, and the subset group of DB with BESG, excluding any possible variation related to different stent grafts. In RFs, the PSV values were higher at every time interval compared to DBs incorporated by BESGs. We can conclude that the elevated velocities in RFs are not related only to the stent type, but rather the general anatomical differences between the 2 types of target artery incorporation is the major factor in velocity differences.

In general, DBs use longer bridging stent grafts sized to the diameter of the vessel (eg, 6-10 mm). This effectively lengthens the bridging stent; as such, the overall resistance of the vessel itself increases. According to Poiseuille’s law, the longer the length of the channel, the greater the resistance, and thus less flow overall, which, all things being equal, would result in reduced velocities.^18^ This is especially evident in comparison to fenestrations, where the stent grafts are usually shorter in length. Tran and colleagues conducted a computational fluid analysis study, which revealed an increase in peak aortic pressures after FEVAR, measured at the aortic inlet. They postulate that this alteration is likely a consequence of the considerable reduction in aortic diameter after the endovascular repair, resulting in an overall increase in resistance.^19^

In another study by Tran et al, it was consistently noted that antegrade branches placed into RAs with an upward orientation experienced a significant decline in branch perfusion as compared to their preoperative values (estimated peak pressure and flow rate). The researchers suggest that this phenomenon is likely caused by the flow being forced down to travel through an extended stent graft with an upward trajectory, resulting in an overall reduction in flow rate compared to the preoperative measurements.^20^ In our study not only renal but also mesenteric arteries showed a significant decrease in PSV in the first 6 to 8 weeks.

We performed a sub-analysis on differences in BESGs and SESGs since these types of stents are 2 distinctly different devices that may affect measured velocities. Since all fenestrated target vessels had BESGs, we only included branched target vessels in our analysis. For mesenteric vessels, BESGs and SESGs seemed to have a significant effect on the PSV measurements (*P* = .011). Although both BESGs and SESGs showed an initial decrease in PSV from baseline to 6 to 8 weeks, BESGs exhibited a greater increase by 6 months (132 ± 46 to 143 ± 47 cm/s) compared to SESGs (119 ± 38 to 126 ± 41cm/s). Over time, PSV in BESGs continued to increase, reaching 198 cm/s by year 5, whereas SESGs demonstrated only modest fluctuations, remaining consistently lower, with a maximum of 136 ± 42 cm/s at year 5. In the RAs, no significant differences were observed between BESGs and SESGs overall (*P* = .78). However, a notable difference was that SESGs showed a significant decrease in PSV from baseline to 6 to 8 weeks (124 ± 61 to 88 ± 47 cm/s, *P* < .001), whereas the decrease in BESGs over the same period was not significant (124 ± 96 to 99 ± 41 cm/s, *P* = .32). This is likely due to the low numbers of branched RAs, as most RAs were incorporated with RFs (n = 72 vs n = 439). The fact that in mesenteric arteries, the BESGs have higher PSV than SESGs could be attributed to the fact that BESGs have a higher radial force compared to SESGs, which are more flexible.^21^

Finally, we reported PSV measurements at the time of secondary interventions for SMAs and RAs. A limitation of this analysis is the small sample. It should be noted that of the 31 vessels requiring a secondary intervention, 11 underwent intraoperative relining with additional covered stents, which may have contributed to an increased risk of restenosis, due to additional stent layers and potential flow disturbance. The median PSV for SMA (n = 3) was 350 cm/s ([IQR], 122-388 cm/s), exceeding the stenosis threshold for native and nonstented vessels (275 cm/s). One of the patients had a velocity below the threshold (122 cm/s), and intervention was indicated based on CTA findings of thrombus at the distal end of the stent. In retrospective analysis at the point of the thrombus, ultrasound measurements were not obtained, and this was a false negative result. The maximum PSV resulted in this case in an underestimation. The median for the RAs was 308 cm/s [IQR, 239-405 cm/s]), which is above the threshold of stenosis for native and nonstented vessels (200cm/s). There were 3 RAs with “normal” velocities (122, 181, and 123 cm/s); however, based on CTA findings they all had high-grade stenosis. Due to the very small number of patients with reintervention of the mesenteric arteries, comparison between DBs and RFs is limited. DBs had lower median PSV for RAs (276 cm/s vs 318 cm/s) as compared to RFs. The decision for secondary interventions was based on both DUS and CTA findings. Of the 24 vessels with a secondary intervention analyzed, 20 vessels (83%) had velocities above the threshold of stenosis for native and nonstented vessels (>200/275 cm/s).^11-13^ When applying DUS criteria for SMA in chronic mesenteric ischemia (CMI), where the threshold for in-stent restenosis is ≥445 cm/s, none of these 3 SMAs with in-stent stenosis would have met this criterion (with PSVs of 388, 350, and 122 cm/s).^22^ This finding highlights the importance of being cautious about extrapolating threshold between patients with atherosclerotic disease and FB-EVAR stenting due to differences in pathophysiology of the underlying abnormality.

The observation that, at some point during follow-up, maximum velocities were above 200 and 275 cm/s in 66 CAs, 36 SMAs, and 38 RAs, without requiring secondary interventions, supports the evidence that only a single PSV threshold based on native vessel criteria should not be the sole reason for secondary intervention. This is further demonstrated by reinterventions in patients with borderline or normal velocity and the wide range of velocities observed in both mesenteric and RAs, independent of the method used for incorporation or the type of bridging stent. Using the DUS criteria for SMA in CMI, where the threshold for in-stent restenosis is ≥445 cm/s, none of the SMAs would have exceeded this threshold. For the CA, where the threshold is ≥289 cm/s, we would have identified 21 cases.^22^ In our experience, the role of ultrasound is to prompt careful evaluation of the CTA to guide decision to intervene keeping in mind that velocities should be considerably high to be the sole reason and normal velocities do not exclude the need for reintervention.

This study strengths include its prospective design, the relatively large number of patients, and the execution of a standardized protocol by experienced sonographers and dedicated RVTs in a single institution. However, there are several limitations to acknowledge. First, the focus was placed on PSV measurements, reflecting the clinical practice, while other possible relevant parameters like renal to aortic ratios and resistive indices were not collected.^11^ Future research should be conducted to investigate these parameters specifically after FB-EVAR. We have chosen to include in the analysis only those arterial segments that would be affected by the presence of the newly placed stent, whether directly (origin and proximal) or indirect velocity jet (mid), so we excluded the distal segments of the target vessels in our analysis, as additional factors such as vessel tapering and branching may account for higher velocities and could be a confounding factor. Another limitation worth mentioning is that we did not examine the effect of tortuosity, angulation, vessel diameter, stent size, or stent length, despite the potential for these factors to influence the PSV values. Additionally, analysis of patients with one or multiple stents was not performed due to the small size of the population with multiple stents. Even though we reported a threshold for stenosis requiring reintervention in RAs, our cohort consisted of only 20 vessels, limiting the value of this threshold and obviating the need for future studies. The small number of patients who underwent secondary intervention for mesenteric vessels limits the ability to define velocity criteria for reintervention. Our study was not designed to identify velocity criteria for reinterventions. This was a descriptive study that did not consider nonintervention cases. Moreover, the comparison between RFs and DBs may be influenced by differences in aneurysm extent (juxtarenal vs TAAA), as these typically correspond to graft designs and anatomical configurations. Finally, the study population gradually decreased in size due to patients being lost to follow-up, while others were added to the timeline, but did not complete all the time marks.

## Conclusion

This longitudinal study demonstrated distinct hemodynamic behaviors between RFs and DBs in both mesenteric and RAs. In mesenteric arteries, RFs showed a significant increase in PSV within the first 6 months, followed by minor, nonsignificant variations. DBs demonstrated an early decline in PSV, with subsequent variations remaining below baseline velocities prior to stent placement. Overall, PSV values for RFs were significantly higher than those for DBs.

In RAs, RFs showed an initial increase at 6 months, followed by a plateau and another significant rise at 5 years. DBs demonstrated an early PSV decline, followed by small nonsignificant variations, consistently remaining below prestent baseline velocities. Similar to mesenteric arteries, RF velocities were higher than DB velocities in RAs. Importantly, despite PSV variations over 5-year follow-up, velocities remained below established thresholds for significant stenosis in native nonstented vessels.

Additionally, in mesenteric arteries, BESGs had higher PSV values over time than SESGs, whereas in RAs, the difference between BESGs and SESGs was not statistically significant. Finally, we have reported PSVs pertaining to stenosis requiring secondary intervention in renal-mesenteric arteries. While the majority of vessels with secondary interventions had velocities above the stenosis threshold, the wide range of velocities observed in both mesenteric and RAs, some of which exceeded thresholds without requiring intervention, suggests that a single PSV threshold should not be the sole criterion for reintervention.

These findings suggested that the type of incorporation and stent selection should be considered in postoperative surveillance and intervention decisions. The reported PSV measurements served as a benchmark for DUS surveillance following FB-EVAR.
